# Induction of immune responses in ducks with a DNA vaccine encoding duck plague virus glycoprotein C

**DOI:** 10.1186/1743-422X-8-214

**Published:** 2011-05-10

**Authors:** Bei Lian, Anchun Cheng, Mingshu Wang, Dekang Zhu, Qihui Luo, Renyong Jia, Fei Liu, Xinfeng Han, Xiaoyue Chen

**Affiliations:** 1 Institute of Preventive Veterinary Medicine, Sichuan Agricultural University, Wenjiang, Chengdu city, Sichuan, 611130, China; 2 Avian Diseases Research Center, College of Veterinary Medicine of Sichuan Agricultural University, Ya'an, Sichuan, 625014, China; 3 Key Laboratory of Animal Diseases and Human Health of Sichuan Province, Sichuan Agricultural University, Wenjiang, Chengdu city, Sichuan, 611130, China

## Abstract

**Background:**

A DNA vaccine expressing glycoprotein C (gC) of duck plague virus (DPV) was evaluated for inducing immunity in ducks. The plasmid encoding gC of DPV was administered via intramuscular (IM) injection and gene gun bombardment.

**Results:**

After immunization by both routes virus-specific serum antibody and T-cell responses developed. Vaccination of ducks by IM injection induced a stronger humoral, but weaker cell-mediated immune response. In contrast, a better cell-mediated immune response was achieved by using a gene gun to deliver DNA-coated gold beads to the epidermis with as little as 6 μg of DNA.

**Conclusions:**

This demonstrated that both routes of DNA inoculation can be used for eliciting virus-specific immune responses. Although DNA vaccine containing DPV gC is effective in both intramuscular injection and gene gun bombardment, the latter could induce significantly higher cell-mediated responses against DPV.

## Background

Duck plague virus (DPV), a member of the Alphaherpesvirinae, is the causative agent of duck plague (DP), one of most serious infectious diseases of waterfowl (duck, geese, and swans)[1,2]. Since the first outbreak in the Netherlands in 1923, this disease has caused heavy economic losses in the commercial duck industry due to mortality, condemnations, and decreased egg production[3,4].

Vaccination is a desirable method to prevent DPV infection. The conventional DPV vaccine are inactivated and attenuated DPV preparations, and they have been shown to be able to confer protection against clinical disease[5,6]. However, as with all or most herpesvirus, DPV has the ability to establish latent infection[7], which adds difficulties in the control and prevention of the transmission of DPV or the establishment of latency. Thus, a more effective therapeutic vaccine will need to elicit sufficient cell-mediated and humoral immune responses.

In recent years, naked DNA encoding immunogenic proteins of infectious agents has been introduced for vaccination. Injection of DNA results in its uptake into cells, expression of the gene and endogenous synthesis of the antigen[8,9]. DNA immunization provides some real advantages over conventional DPV vaccines, including major histocompatibility complex (MHC) class I and II presentation of native antigens, stability, and low production cost[10]. Because DNA vaccination induces a response in both the humoral and cellular arms of the immune system, this approach offers new opportunities in the development of vaccine.

Viral surface glycoproteins are primary targets for immune responses and for the development of viral vaccines. Viral glycoprotein C is one of the several suface glycoproteins present on the mature virus and infected cell membrane. gC is the major target for virus-neutralizing antibodies and has also been reported as the target for T-cell responses. In the cases of pseudorabies (PRV)[11,12], herpes simplex virus type 1 (HSV-1)[13], herpes simplex virus type 2 (HSV-2)[14] and bovine herpesvirus-1 (BHV-1)[15], gC has been shown to induce immunity and provide protection against lethal challenge following DNA immunization.

In the present study, we investigated DPV DNA vaccination in ducks, the natural host of the virus. Vaccination was carried out by injecting ducks with DNA encoding gC of DPV. We also made a comparison between intramuscular (IM) injection and gene gun immunization of the plasmid, and test the efficiency of immunity induced by both routes. Here, we presented evidence that DPV gC DNA vaccine indeed elicited humoral and cell-mediated immune responses in ducks, and gene gun delivery could induce a more potent cell-mediated immune responses compared with the IM route.

## Results

### ConA-induced lymphoproliferation of peripheral blood lymphocytes (PBLs)

**Figure 1:**
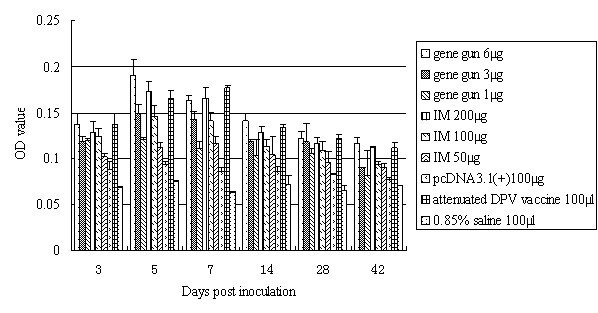
**Lymphoproliferation assay**. Proliferative responses were measured by MTT incorporation assessed as the OD at 570 nm. The results represent three separate experiments (mean OD ± SD).

To analyze the proliferative response, the PBLs were isolated from heparinized blood samples. Figure 1 shows that at 3 dpi, the reaction of T lymphocytes to ConA in PBLs of IM and gene gun immunization groups were obviously higher than groups pcDNA3.1(+) and 0.85% saline (P < 0.05), and achieved peak value at 5-7 dpi. Ducks immunized via gene gun bombardment exhibited significantly higher lymphoproliferation responses than did those immunized intramuscularly (P < 0.05), and the most efficient DNA immunization was achieved by bombarding of skin with DNA-coated particles with the dose of 6 μg plasmid. From our observations, lymphoproliferation responses of 6 μg plasmid via gene gun bombardment was significant higher than 3 μg and 1 μg via the same route between 3 and 42 dpi (P < 0.05), and was significant higher than those injected with 200 μg (5 dpi), 100 μg (between 5 and 42 dpi), and 50 μg (between 3 and 42 dpi) via IM injection (P < 0.05).

The pcDNA3.1(+) naked vector-immunized ducks did not exhibit any detectable lymphoproliferation response and certainly there was no lymphoproliferation activity detected from the negative control group.

### Analysis of T lymphocytes in PBLs

**Figure 2:**
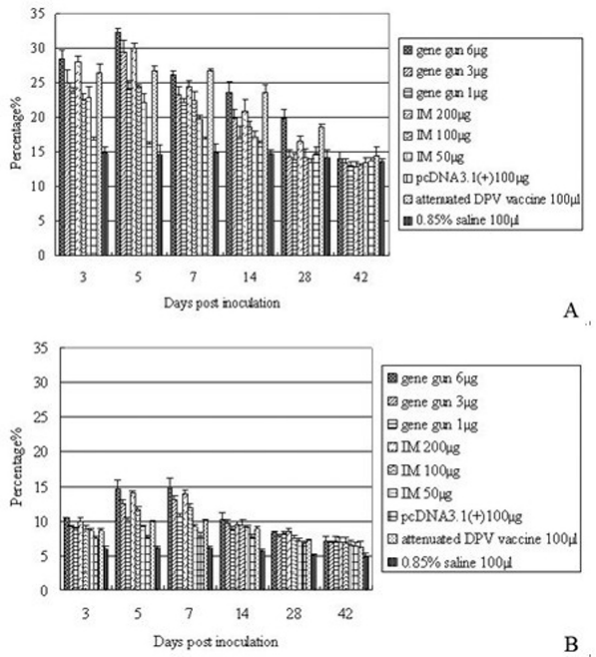
**T lymphocytes in PBLs following DPV gC DNA vaccination**. 3, 5, 7, 14, 28, 42 days after vaccination, the isolated PBLs were stained with monoclonal antibodies against duck CD4 (A), and CD8 (B). The results presented are the mean of all specimens of each group ± SD.

To determine the population of CD4+ and CD8+ T lymphocytes in PBLs, single-cell suspensions of PBLs were prepared following immunization and were examined by flow cytometry using antibodies against CD4 and CD8, FITC-labeled secondary antibodies, and PE, respectively. Figure 2 shows representative data from flow cytometric analysis stained with FITC and PE. Since day 3, the numbers of CD4+ and CD8+ T cells produced by either gene gun or IM injection were significant higher when compared to results for negative and pcDNA3.1(+) naked vector control groups (P < 0.05), and peak levels were reached between 5 and 7 dpi (Figure 2A and 2B).

The strongest induction in CD4+ and CD8+ T cells was observed in 6 μg plasmid via gene gun bombardment at all time points analyzed. The numbers of CD4+ T cells were significant higher than 3 μg and 1 μg via the same route between 3 and 28 dpi (P < 0.05), and were significant higher than those injected with 200, 100 and 50 μg via IM route between 5 and 28 dpi (P < 0.05). The numbers of CD8+ T cells were significant higher than 3 μg (between 3 and 7 dpi) and 1 μg (between 3 and 14 dpi) via gene gun bombardment, and were significant higher than 200 μg (7 dpi), 100 μg (between 3 and 7 dpi) and 50 μg (between 3 and 28 dpi) via IM injection (P < 0.05).

A weaker induction in CD4+ and CD8+ T cells was observed in 200 μg plasmid via IM injection. The numbers of CD4+ T cells were significant higher than other DNA vaccinated groups between 3 and 28 dpi (P < 0.05). The numbers of CD8+ T cells were significant higher than the other two IM groups between 3 and 7 dpi, and were significant higher than 3 μg (3, 5, 28 dpi) and 1 μg (between 3 and 7 dpi) via gene gun bombardment (P < 0.05).

### Antibody responses induced by gene gun immunization or IM injection of gC DNA vaccine

**Figure 3:**
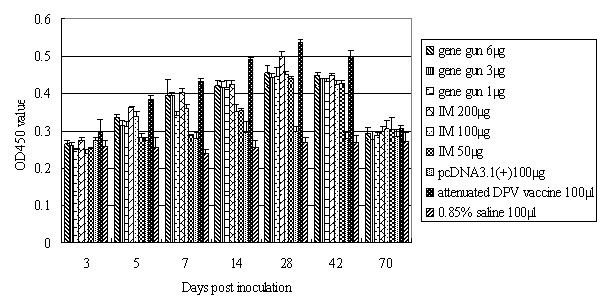
**Serum IgG antibody titers obtained by ELISA assays**. Ducks were immunized with the pcDNA3.1(+)/gC DNA vaccine by IM injection or gene gun bombardment, and serum was collected at days 3, 5, 7, 14, 28, 42, and 70, as indicated. The results represent the average of triplicate wells and are expressed as means ± SD.

Serum samples were taken at indicate times after immunization, and ELISA analysis showed that specific serum IgG titers started to increase 5 days (P < 0.05) after immunization in the DNA vaccinated groups compared with the saline and pcDNA3.1(+) groups. As shown in Figure 3, the serum IgG titers reached a maximum on day 28. Both IM and gene gun groups revealed similar pattern of antibody response. In contrast, no antibody response was observed in the saline group.

Of IM groups, 200 μg plasmid induced the greatest increase in titer of antibody by day 42 after immunization, and this level returned to baseline by day 70. In contrast, 100 μg and 50 μg plasmid did not significantly increase the titer of antibody. The antibody titer of 200 μg IM group was significant different from 100 μg (between 3 and 42 dpi) and 50 μg (between 5 and 28 dpi) IM groups (P < 0.05). On the other hand, of gene gun groups, 6 μg plasmid induced a greater increase in titer of antibody, but this effect was not significant when compared to results for 3 μg and 1 μg gene gun groups (P > 0.05). Comparison of serum IgG titers between IM and gene gun groups revealed that IM injection induced an overall higher antibody titer than gene gun immunization, and 200 μg was optimal for generating high antibody titers in this assay.

### Induction of DPV neutralizing antibodies

Groups of 126 ducks were immunized by DPV gC DNA vaccine, or injected with 0.85% saline, pcDNA3.1(+) and DPV attenuated vaccine as described in materials and methods. 3d, 5d, 7d, 14d, 28d, 42d, 70d after immunization, sera were collected from two randomly chosen ducks per group, and the neutralization titers were determined using a virus neutralization test.

Ducks immunized by IM injection with pcDNA3.1(+) had generated low but detectable levels of neutralizing antibody, but those injected with 100 μl 0.85% saline had no detectable neutralizing antibody responses. In contrast, DNA vaccine immunized groups had generated positive titers at most of the sampling times. The level of neutralization titer in both IM and gene gun immunized groups rose very rapidly since 7 dpi, reached peak levels between 28 and 42 dpi, and then the peak levels decreased by various rates but still had a detectable level at the terminal of experiment (Table 1).

**Table 1:** Changes of neutralizing antibody titer with different inoculation routes and different doses in ducks.

Days post inoculation	Serum neutralizing (SN) titer^a^								
	Immune groups						Control groups		
	Route and dose of inoculation								
	Gene gun bombardment			IM			pcDNA3.1(+) 100 μg	DPV attenuated vaccine 100 μl	0.85% saline 100 μl
	6 μg	3 μg	1 μg	200 μg	100 μg	50 μg			
**3**	1:2.34	1:2.34	<	1:2.34	<	<	<	<	<
**5**	1:2.37	1:2.34	<	1:2.87	1:2.34	<	<	1:3.35	<
**7**	1:2.82	1:2.7	1:2.34	1:3.2	1:2.58	1:2.51	<	1:5.01	<
**14**	1:5.62	1:3.35	1:3.4	1:4.73	1:2.87	1:2.7	1:2.34	1:6.2	<
**28**	1:6.3	1:4.73	1:3.2	1:6.4	1:5.01	1:4.73	1:2.34	1:5.69	<
**42**	1:5.69	1:4.6	1:2.82	1:6.7	1:5.56	1:3.2	<	1:4.73	<
**70**	1:3.4	1:2.73	<	1:3.35	1:2.37	<	<	1:2.82	<

^a^Ducks were immunized via IM or gene gun injection with DPV gC DNA vaccine. The neutralizing antibody titer represents the average of the titers from 3 serum samples standard error. The titer in each group was expressed as the mean ± SD of all specimens in each group.

## Discussion

Immunization of animals with naked DNA encoding protective viral antigens promotes the induction of humoral immune response mimicking natural infection and cell-mediated immune response after intracellular expression of the antigens. However, the effectiveness of a DNA vaccine depends on several factors, such as the selection of viral gene, the route of immunization, and the method of vaccination.

Numerous studies have demonstrated that viral glycoprotein C plays an important role in the induction of antibody and T-cell response to the infection of herpesvirus, and also the protection against lethal challenge[16-21]. In view of such evidence, we constructed a DNA vaccine expressing gC of DPV and evaluated its role in eliciting antibody and T-cell responses in vaccinated ducks.

Intramuscular and intradermal routes have been used by many investigators. Previous experiments have suggested that by direct injection, both muscle and skin have the capacity to take up and express DNA-encoded sequences without a special delivery system among mammalian cells[22,23]. Although IM delivery of DNA has been by far the most studied to date, muscle is not considered to be a site for antigen presentation because it contains few if any dendritic cells, macrophages, and lymphocytes. In contrast, skin has associated lymphoid tissue, comprising Langerhans cells, dendritic cells, keratinocytes and other immune cells, which makes it highly immunocompetent[24]. Among the several techniques of DNA vaccine administration developed to date, gene gun immunization has become highly popular. This technique significantly reduces the amount of plasmid DNA needed for immunization, which propels plasmid-coated gold beads into the skin by pressure and achieves the most efficient DNA immunization[25,26]. So the present study was designed to evaluate the effect of the route of DNA inoculation on vaccination.

Our results demonstrate that both IM and gene gun of administration can be used for DPV gC DNA vaccine. Gene gun delivery of DNA into the epidermis is a very efficient method of inoculation, achieving comparable neutralizing antibody and ELISA antibody with 8-200 times less DNA than direct inoculations of purified DNA in saline (1-6 μg as opposed to 50-200 μg of DNA) (Figure 3). In our tests, both routes mounted a neutralizing antibody and ELISA antibody with high titers in the gC DNA-immunized ducks compared with the saline and pcDNA3.1(+) groups. We think the remarkable success of gene gun vaccination reflects the combination of efficient transfection with efficient antigen presentation and recognition. The gene gun represents a very effective method of transfecting a tissue[27]. When the epidermis is transfected, DNA-expressed antigens are subject to immune surveillance by the skin-associated lymphoid tissue. This lymphoid tissue is rich in cells (such as epidermal Langerhans cells) that are capable of presenting transfected antigens to the T-helper component of the immune system[28].

DNA immunization is thought to induce both humoral and cell-mediated immunity, providing access of endogenously synthesized antigens to the MHC class I- and class II-restricted pathways[29,30]. ConA-induced lymphoproliferation assays of PBLs showed that immune and control ducks showed a non-specific response to ConA, but the DNA-vaccinated groups raised a stronger T lymphocytes proliferative response. Additionally, a better proliferative response in gene gun immunized ducks was detected, possibly due to the more prevalency of antigen-presenting cells (APCs) and higher transfection efficiency in skin than muscle, suggesting that immunization by gene gun bombardment might be a more efficient method for the administration of our DNA construct.

It is the prevalent opinion that the inoculation of naked plasmid DNA elicits Th cell and cytotoxic T lymphocyte (CTL) responses via intramuscular injection or gene gun delivery. In humans and mice, the cellular immune response is predominantly CD4+ and Th1-like after HSV-2 infection[31]. CD4+ T cells are necessary to generate a CD8+ CTL response and viral clearance after infection with HSV[32]. CD8+ T lymphocytes limit infection in the peripheral nervous system, maintain the integrity of neurons during primary HSV infection[33], and resolve HSV lesions[34]. From our present observations, both CD4+ and CD8+ T lymphocytes showed a significant increase in the early stage after immunization. Highest numbers of CD4+ T cells were detected at an earlier time post infection than CD8+ T cells, and a great increase was seen in the CD4+ T cells population, while CD8+ T cells increased to a lesser degree in DNA immunized ducks. The CD4+ T cell response in immunized ducks is substantial and is maintained at a relatively high level by the time point at the terminal of experiment. Similar results were obtained in CD8+ T cells. The strongest induction in CD4+ and CD8+ T cells was observed in 6 μg plasmid via gene gun bombardment at all time points analyzed. These observations, together with the results above, suggest that gene gun immunization, i.e., bombardment of skin with DNA-coated particles, is an efficient method for the administration of DPV gC DNA vaccines. We also noted that cell-mediated immune response occurred earlier than humoral immune response, but the latter is more long-lasting than the former, probably because CD4+ T cells help for B cells maintain a specific antibody response that functions to keep this virus in check.

In summary, this investigation has indicated that vaccination of ducks with a DNA vaccine expressing gC of DPV had the potential to induce antibody production and lymphocyte proliferation, and increase the numbers of CD4+ and CD8+ T cells in PBLs. Both of the two routes of administration involved in this study could raise immune responses, but gene gun bombardment induced a more effective cell-mediate immune response than muscle. However, further studies are needed to identify whether the immunity, which is induced by the vaccine, was sufficient to protect vaccinated ducks completely from DPV challenge.

## Conclusions

In this work, a DNA vaccine encoding gC of DPV was constructed and immunized ducks by IM injection and gene gun bombardment to determine the optimal method of delivery for immune stimulation. Although cell-mediated and humoral immune responses against DPV could generate after immunization by both routes, gene gun bombardment induced a stronger cell-mediated immune response than muscle, suggesting that gene gun delivery may be a more efficient method for the administration of DPV gC DNA vaccine.

## Methods

### Virus and cells

Duck embryo fibroblast (DEF) cells were cultured in minimal essential medium (MEM) (Gibco-BRL) supplemented with 10% fetal bovine serum (FBS) (Gibco-BRL) at 37°C. DPV CH virulent strain was propagated on DEF cells and subjected to titer determination.

### Eukaryotic expression plasmid construction

pcDNA3.1(+)/gC was constructed by inserting the product of gC PCR cloned from the DPV CH virulent strain as described previously[35]. Plasmids were grown in Escherichia coli DH5α and extracted by TIANprep plasmid extraction kit (Tiangen, China) according to manufacturer's instructions. The inserted fragment was confirmed by restriction endonuclease digestion analysis, PCR amplification and DNA sequencing. The pcDNA3.1(+)/gC was transfected in COS-7 cells, and gC expression in COS-7 cells was examined by indirect immunofluorescence test (data not shown).

### Immunization and specimen collection

This study was conducted with 126 Peking ducks (21 days old) from a DPV-free farm which were not vaccinated against DPV. The serum samples of all experimental ducks were tested and found to be DPV antibody negative by ELISA methods and DPV antigen negative by PCR assay[36,37]. A total of 126 ducks were divided into 9 groups, with 14 ducks in each group. Routes of DNA inoculation included intramuscular and gene gun immunization. For intramuscular injection, 200, 100 and 50 μg of DNA was administered in 100 μl of saline. For gene gun delivery, 6, 3 and 1 μg of DNA was administered, and with each shot, 1 μg of DNA immobilized onto 0.5 mg gold particles was delivered at a pressure of 300 pounds per square inch (psi) with a Helios gene gun (Bio-Rad). Within control groups, Group 7 was injected with 100 μg of pcDNA3.1(+), Group 8 was vaccinated with one commercial dose of DPV attenuated vaccine (100 μl) by intramuscular route, and Group 9 was injected with 100 μl 0.85% saline as negative control.

Blood samples from the jugular vein were collected at 3, 5, 7, 14, 28, 42, 70 days post inoculation (dpi). At each of seven sampling times between 3 and 70 dpi, two vaccinated ducks of each group were chosen randomly for sampling.

### Proliferation assays

Peripheral blood lymphocytes (PBLs) were isolated from heparinized blood samples and resuspended at a concentration of 5 × 10^5 ^cells per ml in RPMI 1640 supplemented with 10% fetal bovine serum (FBS), 100 U/ml penicillin, and 100 μg/ml streptomycin. Subsequently, 100 μl volumes of cell suspension were dispensed into 96-well culture plates in triplicate and the PBLs were cultured in the absence or presence of concanavalin A (ConA) (final concentration, 20 μg/ml). After 44 h incubation with 5% CO_2 _at 37°C, 10 μl MTT [3-(4,5-dimethylthiazol-2-yl)-2,5-diphenyltetrazolium bromide] (5 mg/ml) per well was added and incubated for another 4 h. The optical density (OD) was determined in triplicate against a reagent blank at a test wavelength of 570 nm and a reference of 630 nm. The value reflected the viable cell population in each well.

### Flow cytometry

PBLs were isolated as described previously. The cells were washed twice in PBS (0.01 M, pH7.4) and were adjusted to a final concentration of 5 × 10^5 ^cells per ml. Then indirect staining of the cells was carried out as follows: anti-duck CD8 monoclonal antibody and anti-duck CD4 monoclonal antibody (AbD Serotec Ltd, UK) were added into the cells and incubated for 30 min at 4°C in the dark, followed by FITC-and PE-labelled goat anti-mouse IgG (AbD Serotec Ltd, UK). Then the cells were washed with PBS and resuspended in 500 μl PBS, and subsequently subjected to flow cytometric analysis. Viable lymphocytes were gated on the basis of forward and side scatter characteristics, and 10,000 events were analyzed for positive staining with FITC or PE. Data analysis was carried out using BD FACSAria software.

### ELISAs

Polystyrene microtitre plates were coated with 1 μg purified DPV per well. After overnight incubation at 4°C, the plates were blocked in PBS containing 5% bovine serum albumin (BSA) at 37°C for 2 h. After three washes with PBS containing 0.05% Tween 20 (PBS-T), 100 μl per well of individual sera from each group was tested with the determined optimal serum dilution. Plates were incubated for 1 h at 37°C and washed as aforementioned. Later, 100 μl per well of 1:3000 diluted anti-duck horseradish peroxidase (KPL, Gaithersburg, USA) was added and the plates were incubated for 1 h at 37°C. After washing, 100 μl per well of 3,3',5,5'-etramethylbenzidine (TMB) was added. The plates were kept for 30 min at room temperature, and the reaction was stopped with 100 μl per well of 2 M H_2_SO_4_. The OD was measured at 450 nm, using a Bio-Rad Model 860 Plate Reader (Bio-Rad, CA, USA).

### Virus-neutralization assays

Sera were separated from blood samples and heat inactivated at 56°C for 30 min. Serial twofold dilutions of sera were prepared in MEM and then mixed with 100 TCID50 of DPV separately and incubated for 1 h at 37°C. 100 μl of the above mixture was added into monolayer DEF cells and incubated at 37°C for 4 days. The presence of replicating virus in the cells was scored by cytopathic assays. Testing of each sample group included DPV-positive and -negative sera as controls. The virus titer of each specimen was calculated by the Reed-Muench method[38].

### Statistical analysis

The SPSS version 17.0 for Windows XP was used for statistical analysis. Data have been presented in the text and figures as means ± SD. Statistical analysis was done using the one-way ANOVA test and a P value of 0.05 was considered significant.

## Competing interests

The authors declare that they have no competing interests.

## Authors' contributions

BL carried out most of the experiments and drafted the manuscript. AC and MW critically revised the experiment design and the manuscript. DK, QH, RY, FL, XF and XY helped with the experiment. All authors read and approved the final manuscript.
